# Orbital two-channel Kondo effect in epitaxial ferromagnetic *L*1_0_-MnAl films

**DOI:** 10.1038/ncomms10817

**Published:** 2016-02-24

**Authors:** L. J. Zhu, S. H. Nie, P. Xiong, P. Schlottmann, J. H. Zhao

**Affiliations:** 1 State Key Laboratory of Superlattices and Microstructures, Institute of Semiconductors, Chinese Academy of Sciences, PO Box 912, Beijing 100083, China; 2 Institut für Physik, Martin-Luther-Universität Halle-Wittenberg, von-Danckelmann-Platz 3, Halle 06120, Germany; 3 Department of Physics, Florida State University, Tallahassee, Florida 32306, USA

## Abstract

The orbital two-channel Kondo effect displaying exotic non-Fermi liquid behaviour arises in the intricate scenario of two conduction electrons compensating a pseudo-spin-1/2 impurity of two-level system. Despite extensive efforts for several decades, no material system has been clearly identified to exhibit all three transport regimes characteristic of the two-channel Kondo effect in the same sample, leaving the interpretation of the experimental results a subject of debate. Here we present a transport study suggestive of a robust orbital two-channel Kondo effect in epitaxial ferromagnetic *L*1_0_-MnAl films, as evidenced by a magnetic field-independent resistivity upturn with a clear transition from logarithmic- to square-root temperature dependence and deviation from it in three distinct temperature regimes. Our results also provide an experimental indication of the presence of two-channel Kondo physics in a ferromagnet, pointing to considerable robustness of the orbital two-channel Kondo effect even in the presence of spin polarization of the conduction electrons.

## Abstract (web summary)

In metals, electronic scattering from defects by the two-channel Kondo effect is expected to cause deviation from standard low temperature behaviour, however this effect has not been unambiguously shown. Here, the authors present evidence consistent with all transport signatures of the effect in ferromagnetic *L*1_0_-MnAl films.

## Graphical abstract

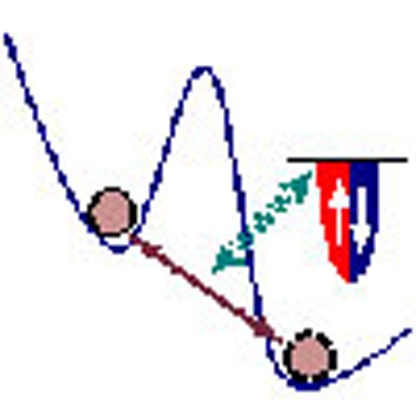

The overscreened two-channel Kondo (2CK) effect displaying exotic non-Fermi liquid (NFL) physics has attracted widespread interest in recent years, especially due to their potential relevance to a host of current topics including strongly correlated physics, Majorana fermions, high-*T*_c_ superconductors, topological matters, carbon nanotubes and quantum dots^1^,^2^,^3^,^4^,^5^,^6^. In spin 2CK effect, a spin-1/2 impurity couples to conduction electrons into two equal orbital channels via an exchange interaction^6^,^7^,^8^,^9^. Below Kondo temperature (*T*_K_) the 2CK model gives rise to impurity quantum criticality accompanied by exotic NFL behaviour as the consequence of two conduction electron spins attempting to compensate the spin-1/2 impurity. However, the strict requirements of zero local magnetic field and channel symmetry make a direct observation of the spin 2CK effect difficult. Intriguingly, an analogous orbital 2CK effect was proposed to arise from resonant scattering centres with orbital degrees of freedom, for example, two-level systems (TLSs)^10^,^11^,^12^. As depicted in Fig. 1a, in a TLS, the tunnelling entity (for example, an atom, atom group or localized electron) coherently tunnels at a rate of 10^8^*–*10^12^ s^−1^ between two independent quantum wells with asymmetry energy Δ_*z*_, tunnelling matrix element Δ_*x*_ and energy splitting Δ=(Δ_*z*_^2^*+*Δ_*x*_^2^)^1/2^ between the lowest two eigenstates^11^,^12^. For the noncommutative model this problem is reduced to the 2CK case, the TLS being represented by a pseudo-spin-1/2 and the spin degeneracy of the conduction electrons being the two channels^10^,^13^. The orbital 2CK effect from TLSs is manifested in electrical transport by a unique temperature (*T*) dependence in resistivity with three distinct *T* regimes (Fig. 1b): a low-*T* upturn characterized by Δ*ρ*_*xx*_∼ln*T* for *T*>*T*_K_, followed by NFL behaviour (Δ*ρ*_*xx*_∼*T*^1/2^) for *T*_D_ (=Δ^2^/*T*_K_)<*T<<T*_K_ and deviation from *T*^1/2^ dependence upon further cooling^11^,^12^. The *T*^1/2^ dependence is a hallmark of the NFL state in the orbital 2CK effect, in striking contrast to the *T*^2^ scaling of Fermi-liquid (FL) behaviour in fully screened Kondo effect^14^. The stability of the low-*T* orbital 2CK fixed point is an important issue. Theoretically, the fixed point is unstable to Δ, channel-symmetry breaking in the exchange coupling and a magnetic field changing the electron population of the two channels, but stable to exchange anisotropy in the Kondo coupling^15^,^16^. Although the TLS model with electron-assisted tunnelling^10^ breaks down in the weak coupling limit^17^,^18^, the 2CK effect is theoretically expected in the case of resonant scattering and strong coupling^19^.

Despite the intensive studies for almost 30 years, the experimental proof for the existence of the orbital 2CK effect has been far from certain. First, it remains a challenge to unambiguously demonstrate the three-regime *T* dependence of the resistivity expected from the orbital 2CK effect in a single material system. Although the NFL behaviour was reported in Cu point contacts (PCs) and glassy ThAsSe single crystal^20^,^21^,^22^,^23^, their small Δ prevented the observation of possible breakdown of the *T*^1/2^ behaviour at lower *T*. Upadhyay *et al*.^24^ observed the NFL behaviour and the low-energy restoration of the FL in conductance spectroscopy of Ti PCs, but there was no indication of a crossover to logarithmic dependence at high energies. Furthermore, the *H* dependence and microscopic mechanisms of the orbital 2CK effect have remained unsettled. Although present theories expect an imbalance in the channel population to produce a crossover to FL behaviour at low temperatures^25^, a magnetic field of up to 14 (5) T did not affect the NFL behaviour in ThAsSe (ref. 22) (Ti PCs^24^). Microscopically, the dynamic tunnelling centres in TLSs were interpreted as a group of nonmagnetic atoms in PCs^20^,^21^, whereas resonant tunnelling of an electron in a polar bond transformation was argued to be responsible for the enhancement of *T*_K_ to a few K in ThAsSe (ref. 22). Therefore, materials with TLSs of larger *T*_K_ and Δ are desirable for a thorough study of the orbital 2CK physics, including its *T* and *H* dependencies and microscopic mechanisms.

Ferromagnetic correlations are predicted to have a detrimental effect on the spin 2CK, while in the case of the orbital 2CK, a coexistence over a large *T* range is possible as the electron spin variable does not directly enter into the interaction process. A crossover to FL behaviour is, however, expected at a low finite temperature in the neighbourhood of the *T*=0 2CK fixed point due to the asymmetry in the channel population. So far, there are two reports on orbital Kondo effect in ferromagnets^26^,^27^. The ferromagnetic UAs_1−*x*_Se_1+*x*_ and Mn_5_Si_3_C_*x*_ display a logarithmic low-*T* upturn in the resistivity. A crossover to FL behaviour was also observed in Mn_5_Si_3_C_*x*_ at low *T*. However, neither shows a *T*^1/2^ dependence characteristic of 2CK effect, possibly because of their low *T*_K_, large *T*_D_ and partial polarization of the conduction electrons.

In this work, we report experimental evidence of TLS-induced orbital 2CK effect in a ferromagnetic system, *L*1_0_-MnAl epitaxial films with strong perpendicular magnetic anisotropy (PMA). We observed a low-*T* resistivity upturn with a clear transition from a ln*T* dependence to NFL behaviour signified by a *T*^1/2^ dependence, and deviation from it upon further cooling. The *T* dependencies are independent of applied magnetic fields up to 8 T. This represents an observation of all three theoretically expected transport regimes from the orbital 2CK effect in the same samples. The greatly enhanced *T*_K_ and Δ in this system suggest fast coherent tunnelling of the TLSs and strong coupling with the conduction electrons. Moreover, the structural disorders in *L*1_0_-ordered films can be tailored by varying the growth parameters^28^,^29^,^30^,^31^,^32^, offering a convenient pathway of tuning the relevant 2CK parameters.

## Results

### Sample structure and ferromagnetism

**Figure 1:**
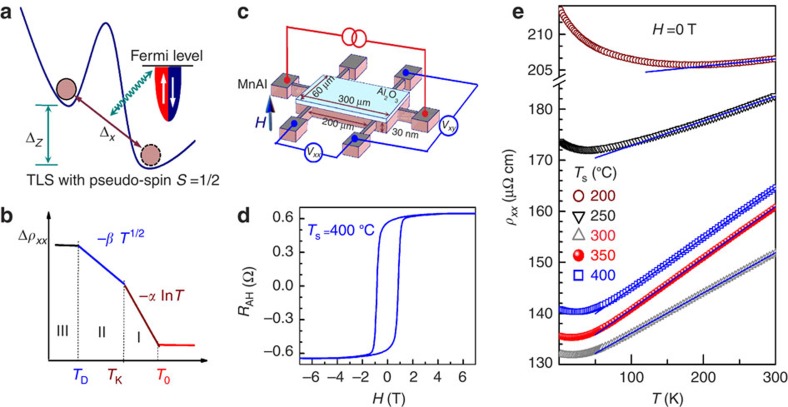
Orbital 2CK effect and typical transport properties. (**a**) Schematic depiction of a TLS with pseudo-spin *S*=1/2 and strong coupling with conduction electrons. Δ_*z*_ and Δ_*x*_ represent the asymmetry energy and tunnelling matrix element of the TLS. (**b**) Expected temperature dependence of Δ*ρ*_*xx*_ for TLS-induced 2CK effect: Δ*ρ*_*xx*_∼−*α*ln*T* for *T*_K_<*T*<*T*_0_ (I), Δ*ρ*_*xx*_∼−*βT*^1/2^ for *T*_D_<*T*<<*T*_K_ (II) and deviation from Δ*ρ*_*xx*_∼−*βT*^1/2^ for *T*<*T*_D_ (III). (**c**) Schematics of the Hall bar device and measurement scheme. The blue arrow represents the direction of the magnetic field. *V*_*xy*_ and *V*_*xx*_ are Hall and longitudinal voltages, respectively. (**d**) Hysteretic anomalous Hall resistance (*R*_AH_) at room temperature for a *L*1_0_-MnAl film with *T*_s_=400 °C. (**e**) *T* dependence of *ρ*_*xx*_ at zero external magnetic field for *L*1_0_-MnAl films with different *T*_s_. The solid lines show the best linear fits at high temperatures, suggesting dominating phonon scattering.

A series of 30-nm-thick *L*1_0_-MnAl single-crystalline films (Fig. 1c) were grown at substrate temperatures (*T*_s_) of 200, 250, 300, 350 and 400 °C. The degree of structural disorder decreases with increasing *T*_s_ from 200 to 350 °C, and then increases when *T*_s_ goes up to 400 °C, which is evidenced by both the intensity and the full width at half maximum of the *L*1_0_-MnAl (002) peaks of X-ray diffraction patterns^33^. These films are strongly strained due to the epitaxial growth as indicated by their shorter *c* axes than bulk (*c*=3.57 Å)^34^, which make them likely candidates to have dynamic defects^24^. These films exhibit strong PMA as revealed by the well-defined hysteretic anomalous Hall resistance (Fig. 1d) and perpendicular magnetization hysteresis^33^.

### Temperature dependence of the longitudinal resistivity

**Figure 2:**
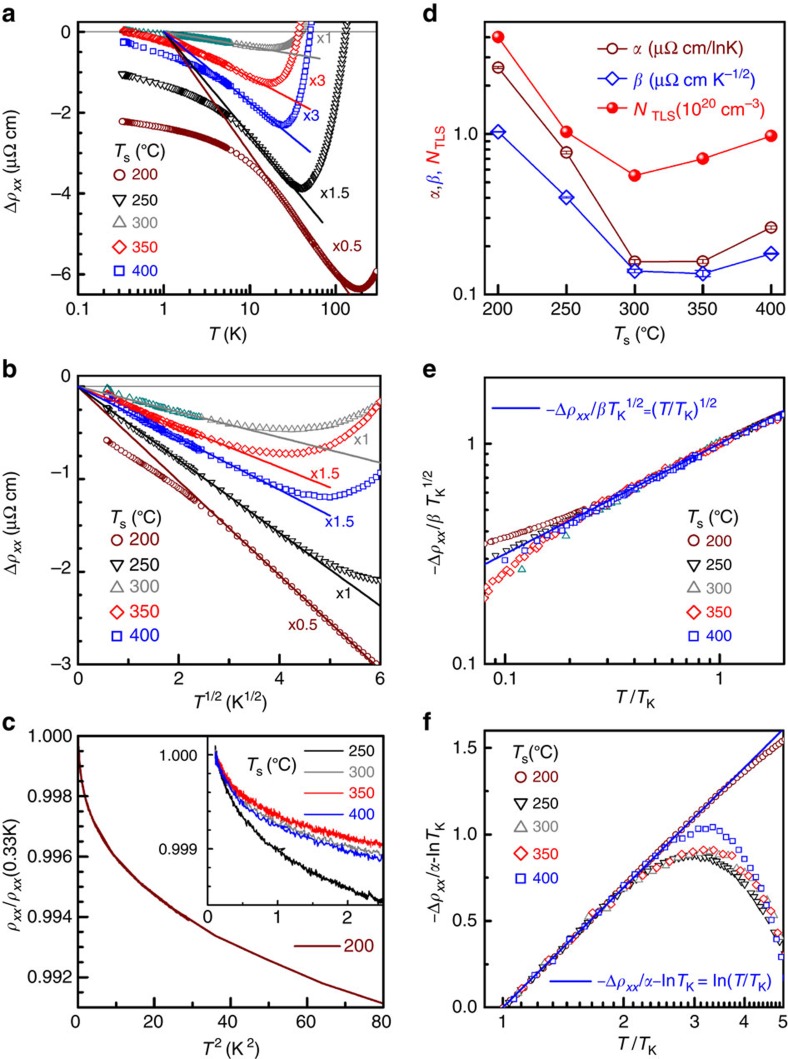
Temperature dependence of zero-field resistivity. (**a**) Semilog plot of Δ*ρ*_*xx*_ versus *T*, (**b**) Δ*ρ*_*xx*_ versus *T*^1/2^, (**c**) *ρ*_*xx*_/*ρ*_*xx*_ (0.33 K) versus *T*^2^, (**d**) *α*, *β* and *N*_TLS_ versus *T*_s_, (**e**) log–log plot of −Δ*ρ*_*xx*_/*βT*_K_^1/2^ versus *T*/*T*_K_ and (**f**) semilog plot of −Δ*ρ*_*xx*_/*α*−ln*T*_K_ versus *T*/*T*_K_ for *L*1_0_-MnAl films grown at different *T*_s_. For clarity, Δ*ρ*_*xx*_ is multiplied by a factor of 0.5 (0.5), 1.5 (1), 1 (1), 3 (1.5) and 3 (1.5) in **a** (**b**) for *T*_s_=200, 250, 300, 350 and 400 °C, respectively. (**e**) and (**f**) are the scaling plots showing the collapse of the experimental data in the respective temperature regimes; the solid blue lines show the equations −Δ*ρ*_*xx*_/*βT*_K_^1/2^=(*T*/*T*_K_)^1/2^ and −Δ*ρ*_*xx*_/*α*-ln*T*_K_=ln(*T*/*T*_K_), respectively.

Figure 1e shows the *T* dependence of the zero-field longitudinal resistivity (*ρ*_*xx*_) of the *L*1_0_-MnAl films. Each film shows a resistivity minimum at a characteristic temperature (*T*_m_) (Supplementary Fig. 1a). In the high *T* regime (*T*>*T*_m_), *ρ*_*xx*_ increases linearly with *T* due to increasing phonon scattering (Supplementary Fig. 1b). Here we show that the low-*T* resistivity upturn in our *L*1_0_-MnAl films likely arises from the TLS-induced orbital 2CK effect. Figure 2a–c plots the *T* dependence of resistivity variation at *H*=0 T for the *L*1_0_-MnAl films, which shows distinct signatures associated with the TLS-induced 2CK effect. In the first regime, as displayed in Fig. 2a, the resistivity increase, Δ*ρ*_*xx*_ (Δ*ρ*_*xx*_=*ρ*_*xx*_−*ρ*_1_, with the offset *ρ*_1_ determined from the best linear fit of *ρ*_*xx*_−ln*T*, see Supplementary Fig. 1c), varies linearly with ln*T* below a temperature *T*_0_ for all films with different *T*_s_, similar to the well-known single-channel Kondo (1CK) effect due to static magnetic impurities^14^. Δ*ρ*_*xx*_ deviates from the ln*T* dependence and transitions to a *T*^1/2^ dependence when *T* drops below *T*_K_ (Supplementary Fig. 2). The *T*^1/2^-dependent resistivity is regarded as a distinct signature of the NFL behaviour from the 2CK effect. Here, an interpretation of localization effects can be excluded, even if a dimensional crossover is considered. Taking into account the resistivity of films (132–214 μΩ cm), which yields a mean-free-path of 2.4–5.5 nm, the film thickness of 30 nm, and the high crossover temperatures (that is, *T*_K_) of up to 23 K, a dimensional crossover seems impossible because neither the thermal length (relevant for e–e interaction) nor the inelastic scattering length (relevant for quantum interference) is likely to approach the film thickness at these temperatures.

**Figure 3:**
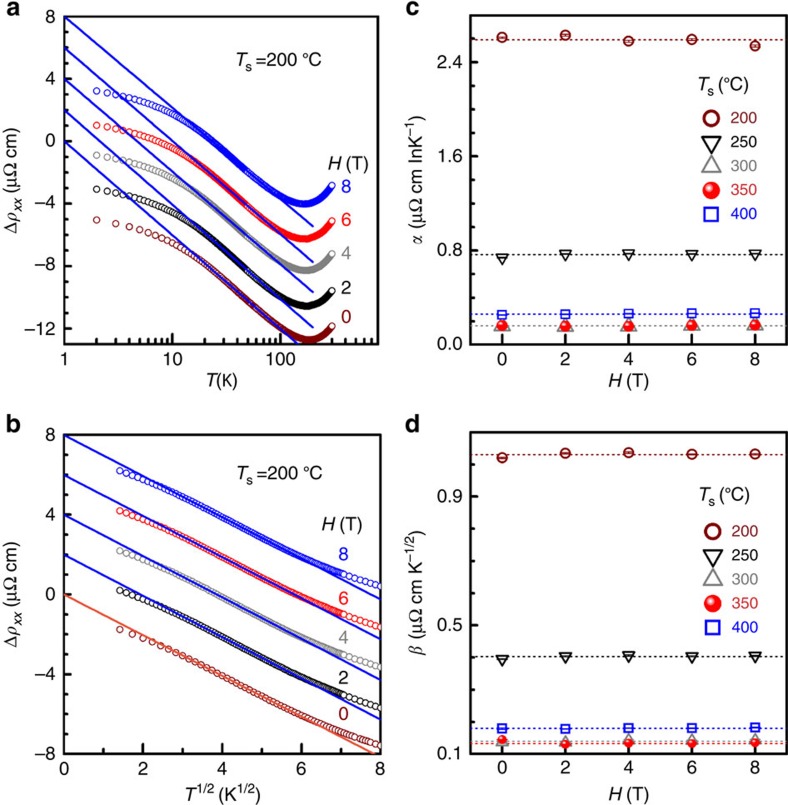
Magnetic field effects on *T* dependence of resistivity. (**a**) Semilog plot of Δ*ρ*_*xx*_ versus *T* and (**b**) Δ*ρ*_*xx*_ versus *T*^1/2^ for *T*_s_=200 °C; (**c**) *α* and (**d**) *β* plotted as a function of *H* for *L*1_0_-MnAl films with different *T*_s_. The magnetic fields (*H*) were applied along the film normal. For clarity, the curves in nonzero fields are artificially shifted by steps of 2 μΩ cm in **a** and **b**. The error bars in **c** and **d** correspond to the mean square root of the statistical error for the best linear fits.

As can be seen in Fig. 2b, Δ*ρ*_*xx*_ (Δ*ρ*_*xx*_=*ρ*_*xx*_−*ρ*_2_, where *ρ*_2_ was determined from the best linear fit of *ρ*_*xx*_−*T*^1/2^; *ρ*_1_ and *ρ*_2_ track each other, see Supplementary Fig. 1c) begins to increase more slowly than *T*^1/2^ below a characteristic temperature *T*_D_, indicating deviation from the NFL behaviour. The deviation is generic, *H*-independent (Fig. 3a) and distinct from that induced by quantum corrections in ThAsSe that only appeared at zero field^22^,^23^. This represents an observation of the TLS theory-expected deviation from the orbital 2CK state below *T*_D_ in a diffusive conductor. We emphasize that the deviation is not due to Joule heating because the resistivities measured under a.c. current of 1 μA and d.c. current of 10 μA are virtually identical. Also, such deviation is not expected from conductance corrections due to electron–electron interaction or quantum interference effects. The lower temperature limit for the *T*^1/2^ regime is given theoretically by *T*_D_=Δ^2^/*T*_K_, from which Δ can be determined. In fully screened 1CK systems^14^ and frozen slow two-state systems^27^,^35^*, ρ*_*xx*_ was observed to saturate following the FL behaviour (∼*T*^2^) at low *T.* The latter have large tunnelling barrier and negligibly small Δ_*x*_ that only allow thermally activated hopping or incoherent tunnelling at a very slow rate (<<10^8^ s^−1^) (ref. 10). However, in the TLS-induced 2CK effect, it has remained unclear how the system experimentally deviates from the NFL behaviour below *T*_D_. To shed light on this important issue in *L*1_0_-MnAl, we measured *ρ*_*xx*_ down to 330 mK. As shown in Fig. 2c, none of these films with different *T*_s_ shows FL-like saturation (*T*^2^) in the studied temperature range, distinctly different from the expectation of a fully screened Kondo effect^14^. There are three possible reasons for this discrepancy: a broad distribution of *T*_D_ of the TLSs; overlapping of the screening clouds of different TLSs; and the spin polarization of the conduction electrons due to the ferromagnetism. A broad distribution of *T*_D_ would severely distort the *T*^1/2^ behaviour and is not a likely scenario. The 2CK displays impurity quantum critical behaviour so that the screening would diverge at *T*=0 under ideal conditions (Δ=0), in contrast to the 1CK problem where the screening radius (*R*) is finite. For orbital 2CK with nonzero Δ, *R* at *T*=0 K limit can be estimated by *k*_F_*R*∼*D*/*T*_D_, where *k*_F_ is Fermi wavevector, *D* is band width (of the order of Fermi Energy *E*_F_). Using *k*_F_ ∼1.7 Å^−1^ (*E*_F_∼11 eV)^36^, *D∼*10 eV, *T*_D_∼1 K for MnAl, one can estimate *R* to be on the order of ∼10^4^ Å at *T*=0 K, which is much larger than the average distance between TLSs (see below). Hence, with decreasing *T* the screening clouds will eventually overlap, although this problem has yet to be quantitatively studied. The spin polarization, if homogeneous, should produce a crossover to a *T*^2^ dependence.

Figure 2d plots the slopes *α*=−*dρ*_*xx*_/*d*(ln*T*) for *T*_K_<*T*<*T*_0_ and *β*=−*dρ*_*xx*_/*d*(*T*^1/2^) for *T*_D_<*T*<*T*_K_ as a function of *T*_s_. It is evident that *α* and *β* have a similar *T*_s_ dependence, that is, *α* (*β*) first drops quickly from their maximum of 2.5 μΩ cm/lnK (1.0 μΩ cm K^−1/2^) at 200 °C to the minimum of 0.2 μΩ cm/lnK^−1^ (0.15 μΩ cm K^−1/2^) at 300–350 °C, and finally goes up to 0.25 μΩ cm/lnK^−1^ (0.18 μΩ cm K^−1/2^) at 400 °C. As is revealed in the metallic PC experiments, thermal annealing can significantly change the number of TLSs^20^,^21^. Here, the non-monotonic dependence of *α* and *β* on *T*_s_ could be ascribed mainly to the thermal tailoring of the density of active TLSs (*N*_TLS_). For the strong coupling TLS centres^11^, *N*_TLS_ can be estimated by 
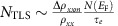
 in the diffusive transport regime, where *Δρ*_*xxm*_, *τ*_e_ and *N*(*E*_F_) are the maximum resistivity upturn due to the TLSs, electron scattering time and density of states at Fermi level, respectively. *τ*_e_ can be determined to be ∼10^−15^ s by *ρ*_*xx*_=m^*^/*ne*^2^*τ*_e_, where m^*^, n and *e* are effective mass, density (*∼*10^22^  cm^−3^) and charge of conduction electrons, respectively. Using a typical *N*(*E*_F_) (ref. 36) of ∼4 × 10^22^ eV^−1^cm^−3^ and experimental values of Δ*ρ*_*xxm*_ (≈Δ*ρ*_*xx*_ at 330 mK), *N*_TLS_ was calculated and shown in Fig. 1d. The fast TLSs were annealed away^20^,^21^ and its density was reduced quickly as *T*_s_ increases to 300 °C; however, the overall population of TLSs increases beyond 350 °C due to the structural deterioration and strain relaxation of the *L*1_0_-MnAl films^33^. The large *N*_TLS_ (∼10^20^ cm^−3^) yields an average distance of ∼20 Å for the TLSs in these films.

The *T*^1/2^ and ln*T* dependencies of the resistivity upturns in different samples can be visualized more directly by collapsing the *T*-dependent Δ*ρ*_*xx*_ data of different samples onto single scaling curves of −Δ*ρ*_*xx*_/*βT*_K_^1/2^=(*T*/*T*_K_)^1/2^ and −Δ*ρ*_*xx*_/*α*−ln*T*_K_=ln(*T*/*T*_K_) for *T*_D_/*T*_K_<*T*/*T*_K_<1 and 1<*T*/*T*_K_<*T*_0_/*T*_K_, respectively. Figure 2e shows the log–log plot of −Δ*ρ*_*xx*_/*βT*_K_^1/2^ against *T*/*T*_K_ for the *L*1_0_-MnAl samples with different *T*_s_, from which log(−Δ*ρ*_*xx*_/*βT*_K_^1/2^) is shown to scale linearly with log(*T*/*T*_K_) with a slope of 1/2 in *T*_D_/*T*_K_<*T*/*T*_K_<1 for all the samples. Similarly, the data for all the samples in the *T* range 1<*T*/*T*_K_<*T*_0_/*T*_K_ can be scaled onto a single curve of −Δ*ρ*_*xx*_/*α*−ln*T*_K_=ln(*T*/*T*_K_), as demonstrated by the semilog plot of −Δ*ρ*_*xx*_/*α*−ln*T*_K_ against *T*/*T*_K_ (Fig. 2f).

### Magnetic field effects

**Figure 4:**
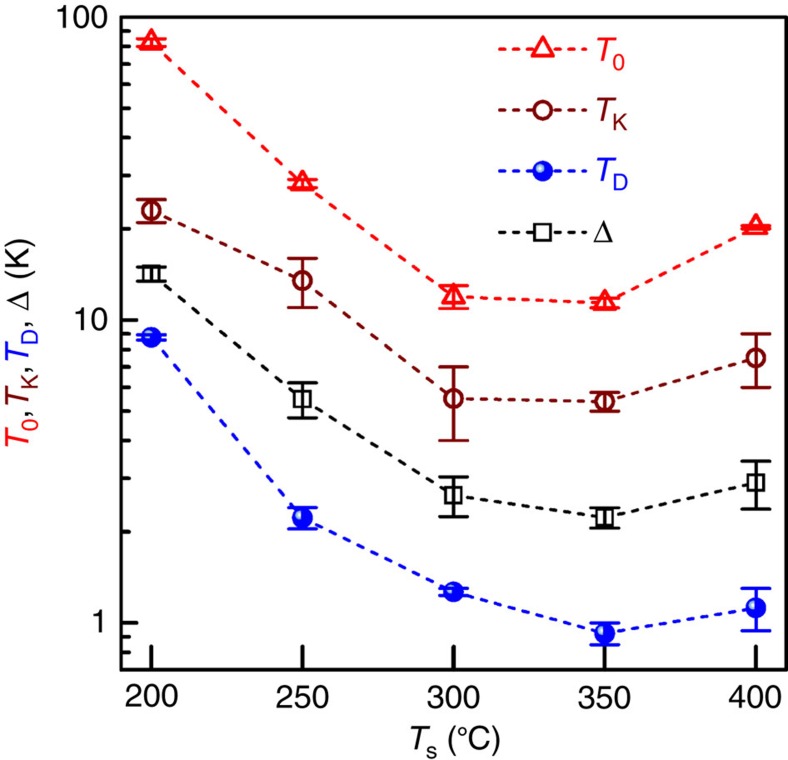
Characteristic temperatures of orbital 2CK effect. *T*_0_, *T*_K_, *T*_D_ and Δ of the *L*1_0_-MnAl films are tuned by varying *T*_s_ of the epitaxial growth. The error bars correspond to the uncertainty due to the overlapping of the adjacent temperature regimes.

To establish more rigorously the orbital 2CK effect in our *L*1_0_-MnAl films, we examined the effect of applied perpendicular magnetic fields, *H*, on the *T*-dependent resistivity. Here, in these films with strong PMA, anisotropic magnetoresistance (MR) and MR from spin disorder scattering under perpendicular *H* should be negligible due to the orthogonal magnetization-current relation and the large energy gap in spin wave excitation spectrum. This is highly amenable to study the *H* dependence of a 2CK effect. As an example, we show *ρ*_*xx*_ (*T*) of the *L*1_0_-MnAl film grown at 200 °C under various *H* from 0 to 8 T in Fig. 3a,b. The magnetic fields have no measurable effects on the *T* dependence: *ρ*_*xx*_ scales linearly with ln*T* and *T*^1/2^ at *T*_K_<*T*<*T*_0_ and *T*_D_<*T*<*T*_K_ (*T*_0_∼82.5 K, *T*_K_∼23 K and *T*_D_∼8.8 K), respectively. The same features hold for other films with different *T*_s_. Figure 3c,d summarizes the values of *α* and *β* as a function of *H* for the *L*1_0_-MnAl films with different *T*_s_. It is clear that both *α* and *β* for all films are independent of *H*, strongly suggesting a nonmagnetic origin of the resistivity upturn scaling in the *L*1_0_-MnAl. Specifically, there is no measurable change in *T*_0_, *T*_K_ and *T*_D_ under different *H* (Fig. 3a,b), suggesting a negligible effect of *H* on the coupling strength, the tunnelling symmetry and barrier height of the TLSs. These observations provide strong evidence for the orbital 2CK effect being induced by TLSs of nonmagnetic impurities. Here, it also should be pointed out that the Zeeman energy (∼0.9 meV at *H*=8 T) is negligibly small in comparison with *E*_F_ and ferromagnetic exchange splitting (*E*_exchange_∼2 eV) in *L*1_0_-MnAl (ref. 36), hence should not have any measurable effect on the channel asymmetry (Δ*N*=*N*_↑_−*N*_↓_, where *N*_↑_ and *N*_↓_ are the numbers of majority and minority spins in the conduction band, respectively) and the three-regime resistivity upturn^16^,^24^,^25^. A small negative MR (<0.5%) is observed in the *L*1_0_-MnAl at high *H* in a broad *T* range from 2 to 300 K, see Supplementary Fig. 3. The MR does not saturate even at 7 T and shows a linear scaling with *H*^1/2^, which is similar to that in ThAsSe (ref. 20). The MR shows a *T* dependence, which appears to have no correlation with any characteristic temperatures of the 2CK effect (Fig. 4). Though not yet well understood, such a MR behaviour in materials showing orbital 2CK effect due to TLSs is unlikely to be related to the *T*^1/2^ dependence of *ρ*_*xx*_ at *T*_D_<*T*<*T*_K_.

### Characteristic temperatures of orbital 2CK effect

Figure 4 summarizes the relevant characteristic temperatures *T*_0_, *T*_K_ and *T*_D_, and Δ of the *L*1_0_-MnAl films as a function of *T*_s_. As *T*_s_ increases, *T*_0_ drops markedly from 82.5 to 11.4 K, and goes up to 20.0 K as a consequence of the non-monotonic dependence of the TLS population on *T*_s_ (Fig. 2d). *T*_K_ represents the temperature below which conduction electrons can overscreen the pseudo-spin of the impurity in an orbital 2CK system. Since there is an overlap between the two *T* regimes of ln*T* and *T*^1/2^ dependencies, the values of *T*_K_ are defined as the centre of the *T* overlap. In striking contrast to the small experimental values in Cu PCs (0.1–5 K) and theoretical calculations on amorphous systems (<<1 K), *T*_K_ ranges from 23.0 to 5.4 K in the *L*1_0_-MnAl. The significant enhancement of *T*_K_ in this system could be understood in terms of resonant scattering due to strong coupling of conduction electrons to the scattering centres^19^. Within this scenario, the decrease of *T*_K_ at higher *T*_s_ may come from a reduced strength of the resonant scattering. Another intriguing observation is that Δ is tuned by as much as a factor of ∼5.6 when *T*_s_ varies between 200 and 400 °C, which is difficult to achieve in previously studied systems of metallic PCs or glasses using a voltage or magnetic field^20^,^21^,^22^,^23^,^24^. As *T*_s_ goes up, the fastest TLSs are annealed away, leaving the slower ones to dominate the scattering of conduction electrons. Therefore, with *T*_s_ increasing from 200 to 350 °C, the value of Δ for active TLSs decreases, leading to a reduction of *T*_D_. The upturn of *T*_0_, *T*_K_ and *T*_D_, and Δ at *T*_s_ of 400 °C may be attributed to the increased population of fast TLSs and enhanced resonant scattering of conduction electrons due to the structural degradation^31^. Here, it is worth mentioning that the values of Δ observed in *L*1_0_-MnAl films are much larger than those reported in Cu PCs (*∼*1 K)^20^,^21^ and ThAsSe (<0.01 K)^22^,^23^. Besides the small tunnelling barriers and fast scattering rates (that is, intrinsic TLS energy splitting), the adjacent Kondo cloud overlaps suggested by the non-*T*^2^ deviation and the ferromagnetic exchange splitting could also be responsible for the great enhancement in the effective values of Δ in these *L*1_0_-MnAl ferromagnetic films. As suggested by the close correlation between the *T*_s_ dependence of the characteristic parameters of the TLS-induced 2CK effect, that is, *α*, *β*, *T*_0_ and *T*_K_, and Δ with those of structural imperfections and the magnetic properties (for example, magnetization and PMA)^33^, electrons as the tunnelling centres of the TLSs in the *L*1_0_-MnAl films can be excluded. Similarly, the magnetic Mn atoms can also be ruled out because of the nonmagnetic nature of the orbital Kondo effect. Therefore, we surmise that the nonmagnetic small Al atoms have the role of TLS centres in the present films. In nanoscale tunnelling systems (for example, 3–10 nm-wide Cu PCs^37^), it has been possible to observe individual constituent of the 1/*f* noise, for example, that results from the motion of atomic defects or electrons. However, such constituent is much more difficult to resolve in metallic films^37^, for example, our *L*1_0_-MnAl epitaxial films. New spectroscopic probe techniques need developing for definitely identifying the microscopic nature of the TLSs in these diffusive metal films.

### Coexistence of orbital 2CK with ferromagnetism

**Figure 5:**
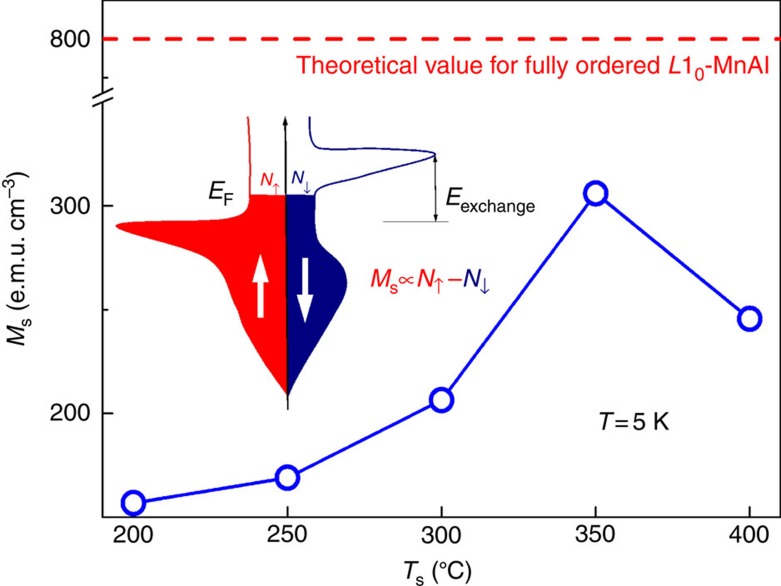
*T*_s_ dependence of saturation magnetization. The red dashed line shows the theoretical value of *M*_s_,that is, 800 e.m.u. cm^−3^, for fully ordered *L*1_0_-MnAl, which is much larger than that at 5 K in the *L*1_0_-MnAl films with different *T*_s_. Inset: a schematic of the partial density of states of a ferromagnet, where *E*_F_, *E*_exchange_ and *N*_↑_ (*N*_↓_) are the Fermi energy, the exchange splitting and the number of the majority (minority) spin in conduction band, respectively.

Now, we discuss the apparent coexistence of orbital 2CK with ferromagnetism. In a conventional ferromagnet, although the Kondo coupling between the TLS and itinerant electrons is irrelevant to the electron spins, the symmetry of the two spin channels is broken due to ferromagnetic exchange splitting of the *d*-band. The channel asymmetry should lead to different tunnelling rates of a TLS for two spin channels and thus weaken the NFL behaviours in comparison with its nonmagnetic counterpart (Δ*N=*0). If the degree of channel asymmetry (*P*=Δ*N*/(*N*_↑_+*N*_↓_)) is large enough, this should be manifested as a decreased magnitude (*β*) and an enhanced effective breakdown temperature (*T*_D_) of the 2CK effect as a result of the enhanced decoupling of TLS from one spin channel. On the basis of a simple assumption in the Stoner model for itinerant ferromagnetism^38^, the saturation magnetization (*M*_s_) can be an index of the channel asymmetry, that is, *M*_s_∝Δ*N.* However, this effect is difficult to quantify experimentally as one cannot vary *M*_s_ while keeping other parameters (for example, *N*_TLS_, coupling strength, Kondo cloud overlap, barrier height and tunnelling rate) of the TLSs constant in a set of samples. For our *L*1_0_-MnAl films, the *T*_s_ dependence of the measured *β* and *T*_D_ is dominated by the variation of the other parameters, respectively, rather than that of Δ*N*, as suggested by the quite different *T*_s_ dependence of the 2CK parameters (*β* and *T*_D_) and *M*_s_. The fully ordered *L*1_0_-MnAl is theoretically an itinerant magnet with a spin polarization of 23.8% at the Fermi surface^39^ and a magnetization *M*_s_ of ∼800 e.m.u. cm^−3^ (that is, 2.37 *μ*_B_ f.u.^−1^)^28^,^36^. In our disordered *L*1_0_-MnAl samples, the measured *M*_s_ is much smaller (157–306 e.m.u. cm^−3^, see Fig. 5), suggesting a very low Δ*N* (<<*N*_↓_) and *P* (<<1). This could be a reason why it is still difficult to establish a definitive quantitative correlation between Δ*N* and the 2CK parameters (*β* and *T*_D_).

## Discussion

We have observed transport behaviour highly suggestive of a robust orbital 2CK effect from TLSs in epitaxial *L*1_0_-MnAl films with strong PMA. The *H*-independent resistivity upturn scaling with ln*T* and *T*^1/2^ in the two *T* regimes below the resistance minimum, and deviation from the NFL behaviour at the lowest temperatures are consistent with the TLS model. The molecular beam epitaxy growth method afforded unprecedented tunability of the TLS density, *T*_K_ and Δ in this material system, leading to the insight on the origin of the 2CK effect. The greatly enhanced *T*_K_ and Δ suggest resonant tunnelling of TLSs due to the strong coupling with conduction electrons. The orbital 2CK effect in a material with significant ferromagnetism and conduction spin polarization is an intriguing observation that warrants further theoretical and experimental studies.

## Methods

### Sample growth and characterization

*L*1_0_-MnAl films were grown on 150 nm GaAs-buffered semi-insulating GaAs (001) by molecular beam epitaxy and capped with a ∼4 nm-thick Al_2_O_3_ layer. The thickness and Mn/Al atomic ratio of *L*1_0_-MnAl layer is calibrated to be 30 nm and 1.1 by cross-sectional high-resolution transmission microscopy with energy dispersive X-ray spectroscopy^31^, respectively. Synchrotron X-ray diffraction and Quantum Design SQUID were used to characterize the structures and magnetization properties of these films.

### Device fabrication and transport measurement

These films were patterned into 60 μm wide Hall bars with adjacent electrode distance of 200 μm using photolithography and ion-beam etching for transport measurements (Fig. 1c). The longitudinal resistivity was measured as a function of temperature (*T*) and magnetic field (*H*) in a Quantum Design PPMS for *T*=2–300 K (DC, *I*=10 μA) and in an Oxford ^3^He cryostat for *T*=0.33–5 K (a.c, *I*=1 μA).

## Additional information

**How to cite this article:** Zhu, L. J. *et al*. Orbital two-channel Kondo effect in epitaxial ferromagnetic *L*1_0_-MnAl films. *Nat. Commun.* 7:10817 doi: 10.1038/ncomms10817 (2016).

## Supplementary Material

Supplementary Information Supplementary Figures 1-3 and Supplementary Reference
