# Supplementary material for: Orbital two-channel Kondo effect in epitaxial ferromagnetic L10-MnAl films
Source: Nat Commun. 2016 Feb 24;7:10817. doi: 10.1038/ncomms10817 (PMC4770089; doi:10.1038/ncomms10817)
Supplement: Supplementary Information — Supplementary Figures 1-3 and Supplementary Reference [file ncomms10817-s1.pdf]

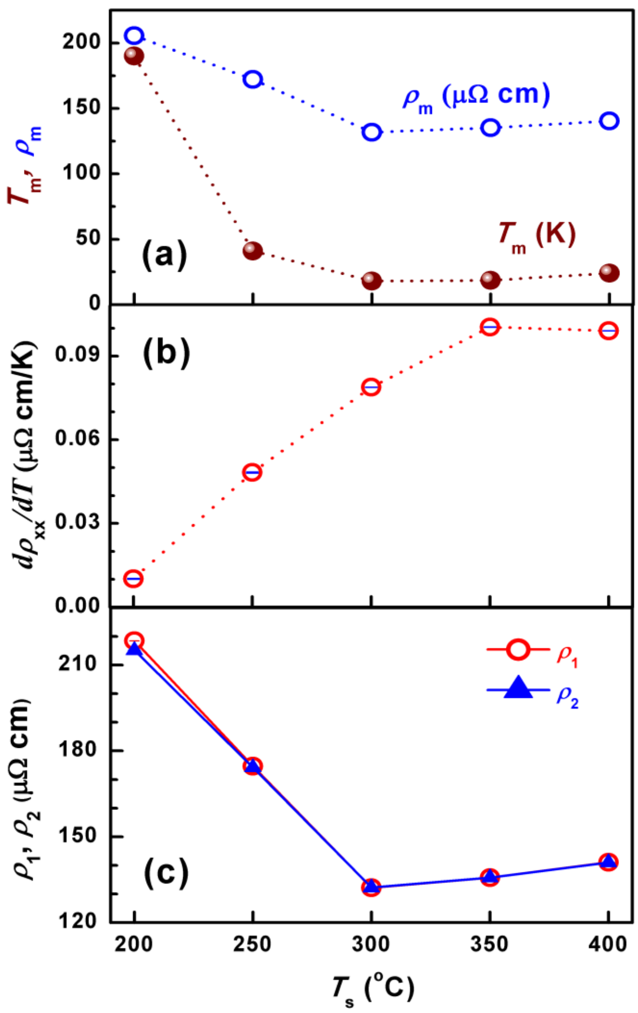

Supplementary Figure 1| Electrical transport of $L1_0$-MnAl films at zero magnetic field. (**a**) Resistivity minimum (ρ_m) and the temperature (T_m) where the minimum occurs. With increasing T_s, T_m moves from 190 K down to 18 K, and then up to 25 K; concomitantly ρ_m drops from 205.7 μΩ cm to 135.3 μΩ cm, and then climbs up to 140.3 μΩ cm. (**b**) The slope $d\rho_{xx}/dT$ in the high T regime ($T > T_m$) where ρ_{xx} increases linearly with T due to increasing phonon scattering. $d\rho_{xx}/dT$ indicates that phonon scattering is significantly enhanced with increasing T_s and reaches a peak at 350 °C. Notably, the drop in $d\rho_{xx}/dT$ and the increases in T_m and ρ_m at T_s = 400 °C appear to be correlated and can be attributed to the deterioration of structural integrity, which is also corroborated by the decreases of the magnetization, coercivity, and PMA of films grown at $T_s \geq 350$ °C [1]. (**c**) The offsets ρ_1 and ρ_2 determined by the best linear fits of $\rho_{xx} = \rho_1 - \alpha \ln T$ for $T_K < T < T_0$ and $\rho_{xx} = \rho_2 - \beta T^{1/2}$ for $T_D < T < T_K$, respectively. ρ_1 and ρ_2 are almost identical for all the samples, indicating negligible variation of the resistivity due to phonon scattering below T_0. The error bars in (b) and (c) are mean square root of the statistical error for the best linear fits.

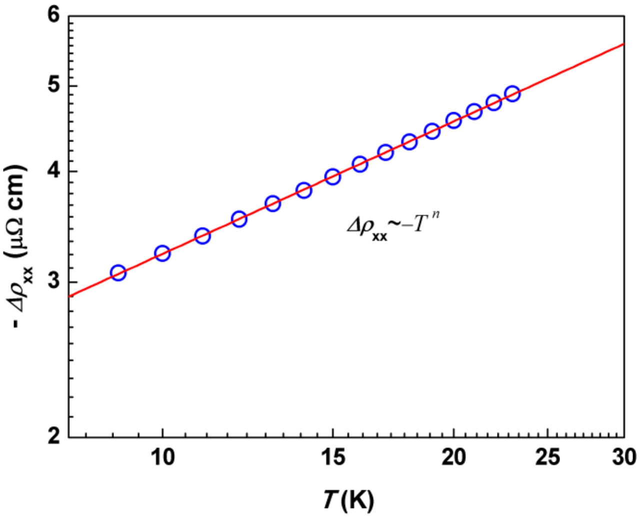

Supplementary Figure 2| Log-log plot of -$\Delta\rho_{xx}$ as a function of T in the $L1_0$-MnAl film grown at 200 °C. The red line represents the best fitting of $\Delta\rho_{xx}$ ~ -T^n in the non-Fermi-liquid regime (T_D<T<T_K), which yields an exponent n of 0.499±0.001, consistent with the expected value of n=1/2 in the 2CK model. Within the overall three-regime resistivity upturn, this provides strong evidence in favor of a 2CK interpretation of the data.

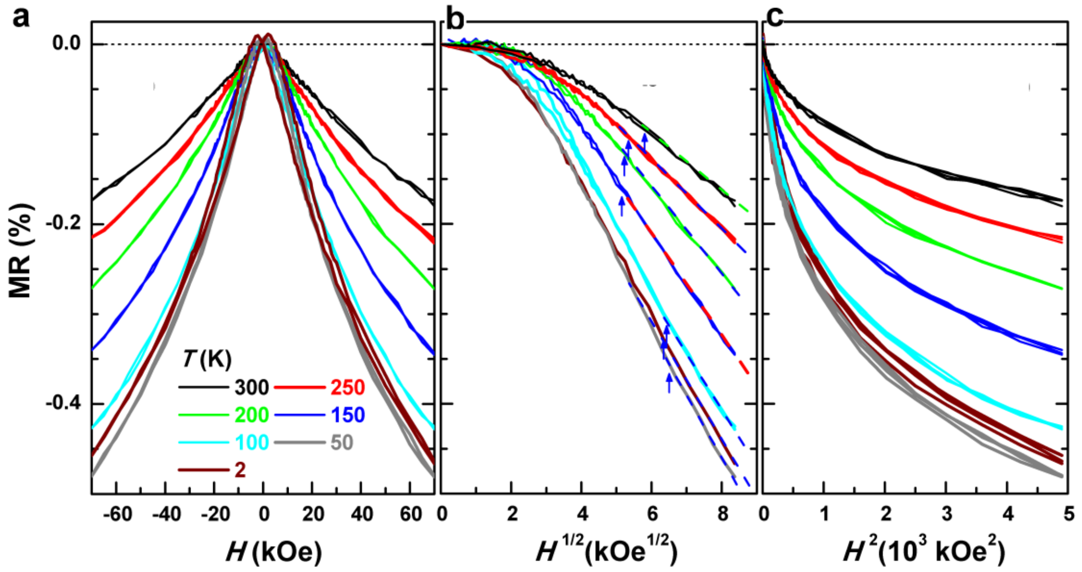

Supplementary Figure 3| Temperature-dependence of magnetoresistance of a $L1_0$-MnAl film grown at T_s=200 °C. (**a**) MR versus H. At low magnetic fields, there is a very small positive MR (<0.013%) for each temperature, which may be attributed to the domain-wall MR or anisotropic MR closely related to magnetization switching. A negative MR appears at high fields for each temperature. (**b**) MR versus $H^{1/2}$. The dashed straight lines are linear fits at high magnetic fields, and the blue arrows represent the magnetic field below which MR deviates from the $H^{1/2}$ scaling. The good linearity at high fields indicates that the negative MR scales with $H^{1/2}$. (**c**) MR versus H^2. The MR-H^2 plots show strong nonlinearity, which is different from magnetic single-channel Kondo effect. Although its detailed mechanisms remain unknown, the observed negative MR in these $L1_0$-MnAl films cannot be attributed to the orbital 2CK effect or two-level systems: First of all, it appears in the broad temperature regime from 300 K to 2 K, instead of the low temperature regime where orbital two-channel Kondo effect occurs. Secondly, it shows no apparent relation with the characteristic temperatures of the orbital two-channel Kondo effect. Finally, it does not show any indication of saturation even at 7 T, which is not consistent with the nonmagnetic origin of the orbital 2CK effect.

Supplementary Reference

1 Nie, S. H., Zhu, L. J., Pan, D., Lu, J., & Zhao, J. H. Structural characterization and magnetic properties of perpendicularly magnetized MnAl films grown by molecular-beam epitaxy. *Acta. Phys. Sin.* **62**, 178103 (2013).
